# Constructing Fluorogenic *Bacillus* Spores (F-Spores) via Hydrophobic Decoration of Coat Proteins

**DOI:** 10.1371/journal.pone.0009283

**Published:** 2010-02-19

**Authors:** Linda Ferencko, Boris Rotman

**Affiliations:** 1 Department of Molecular Biology, Cell Biology and Biochemistry, Brown University, Providence, Rhode Island, United States of America; 2 BCR Diagnostics, Jamestown, Rhode Island, United States of America; Texas A&M University, United States of America

## Abstract

**Background:**

Bacterial spores are protected by a coat consisting of about 60 different proteins assembled as a biochemically complex structure with intriguing morphological and mechanical properties. Historically, the coat has been considered a static structure providing rigidity and mainly acting as a sieve to exclude exogenous large toxic molecules, such as lytic enzymes. Over recent years, however, new information about the coat's architecture and function have emerged from experiments using innovative tools such as automated scanning microscopy, and high resolution atomic force microscopy.

**Principal Findings:**

Using thin-section electron microscopy, we found that the coat of *Bacillus* spores has topologically specific proteins forming a layer that is identifiable because it spontaneously becomes decorated with hydrophobic fluorogenic probes from the milieu. Moreover, spores with decorated coat proteins (termed F-spores) have the unexpected attribute of responding to external germination signals by generating intense fluorescence. Fluorescence data from diverse experimental designs, including F-spores constructed from five different *Bacilli* species, indicated that the fluorogenic ability of F-spores is under control of a putative germination-dependent mechanism.

**Conclusions:**

This work uncovers a novel attribute of spore-coat proteins that we exploited to decorate a specific layer imparting germination-dependent fluorogenicity to F-spores. We expect that F-spores will provide a model system to gain new insights into structure/function dynamics of spore-coat proteins.

## Introduction

*Bacilli* and *Clostridia* have a two-stage lifecycle in which growing bacteria in response to nutritional deprivation undergo an elaborate developmental program leading to spore formation. Spores play critical roles in long term survival of the species because they are highly resistant to extreme environmental conditions and also capable of remaining metabolically dormant for years. Despite their ruggedness and extreme longevity, spores rapidly respond to the presence of small specific molecules known as germinants that signal favorable conditions for breaking dormancy through germination, an initial step in the process of completing the lifecycle by returning to vegetative bacteria. Early molecular events triggering germination have remained an elusive target partly because they include a complex cascade of biochemical and structural changes that take place without any apparent energy source (see [1], [2] for recent reviews).

The spore's exceptional resistance is attributed to its distinctive morphology consisting of three concentric separate compartments: the core, cortex, and coat. At the center, the core houses the DNA and RNA and is encased by the cortex, a thick peptidoglycan layer, which in turn, is surrounded by the coat, a multilayer assembly of heterogeneous proteins [3], [4], [5].

Historically, the coat has been considered a static structure providing rigidity and mainly acting as a sieve to exclude exogenous large toxic molecules, such as lytic enzymes. Over recent years, however, new information about the coat's architecture and function have emerged from experiments using innovative tools such as automated scanning microscopy [6] and high resolution atomic force microscopy [7], [8]. At present, the coat is regarded as a mechanically flexible structure capable of undergoing rapid volume expansion and contraction without any apparent effect on the dormancy of spores [6], [9]. Considering this remarkable dynamism in the context of the coat's elaborate biogenesis [5], convoluted surface morphology [7], [8], [10] and network of about 60 different proteins [11], [12], it seems reasonable to assume that other novel attributes and functions of the coat are yet to be uncovered [3].

Here we report a previously unrecognized physiological property of the coat in dormant *Bacillus* spores from different species. Namely, spores exposed to hydrophobic fluorogenic probes—such as fluorescein acyl esters and nucleic acid stains of the Syto family—spontaneously use the probes to decorate coat proteins forming a well-defined layer that is clearly distinguishable under thin-section electron microscopy (TEM). In addition, we found that spores with decorated layers (termed F-spores) are fluorogenic, i.e., they generate intense green fluorescence upon germination. As described below, data from different lines of experimentation show that the fluorogenic ability of F-spores is under control of the germination apparatus. Altogether, our results indicate that F-spores have potential as tools for studying germination-dependent dynamic changes of coat proteins.

## Results

### Topological Specificity and Quantification of Decorated Layers in F-Spores

**Figure 1:**
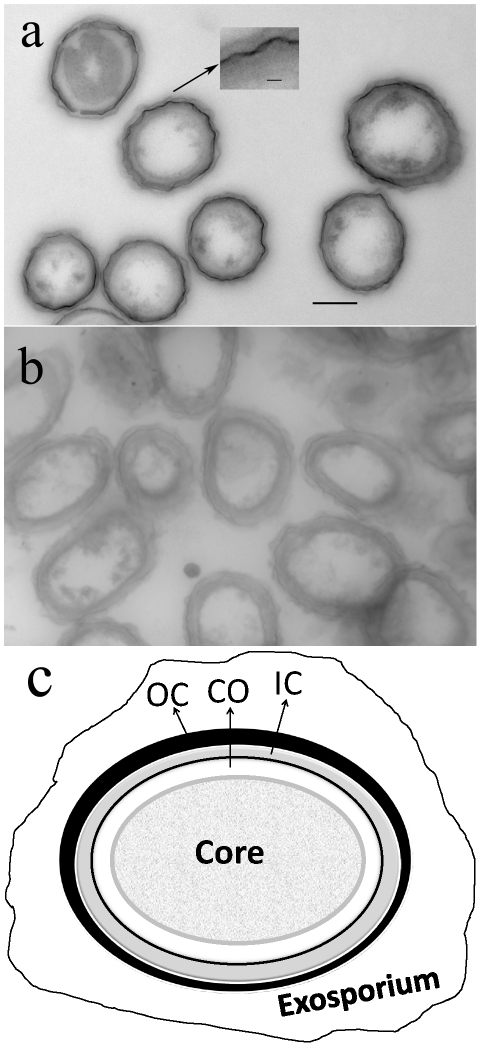
Topologically specific electron-dense layer in F-spores. Electron micrographs of unstained ultrathin (60 nm) cryosections. (*a*) F-spores from *B. megaterium* constructed with diacetyl-2,4,5,7-tetraiodo-fluorescein, an electron-dense fluorogenic probe. A conspicuous dark layer of about 14-nm thickness indicates binding of the high electron density probe to topologically specific coat proteins.* (*b*) Normal spores. (*c*) Line drawing representing a spore cross section: OC, outer coat; IC, inner coat; CO: cortex. Scale bars: 500 nm (*a–b*) and 40 nm (*a Inset*).

Decorated layers were visualized by TEM using 60-nm cryosections of *B megaterium* F-spores constructed with diacetyl-2,4,5,7-tetraiodo-fluorescein, an electron-dense fluorogenic substrate of esterases. Cryosectioning was essential for two reasons: first, to avoid use of organic solvents causing loss of hydrophobic fluorogenic substrates; and second, to circumvent staining of the outer coat proteins with heavy metals, such as lead, osmium and uranium, normally used for conventional TEM. Therefore, we used a cryosectioning method employing reagents devoid of electron-dense atoms. As illustrated in Fig. 1*a*, cryosections of F-spores show a clearly distinguishable electron-dense layer of about 14-nm thickness attributable to binding of diacetyl-2,4,5,7-tetraiodo-fluorescein because no similar layer is present in dormant spores (Fig. 1*b*). The electron-dense layer appears to be located in the coat near the spore surface (Fig. 1*a* inset), although a location in the exosporium near the coat cannot be excluded because the exosporium may not be visible under these conditions. In contrast to F-spores, cryosections of normal spores show a broader, uniform layer of reduced electron density (Fig. 1*b*).

Within the context of these results, it should be mentioned that electron micrographs obtained by conventional TEM show an electron-dense layer in the outer coat, which is the result of heavy metal staining [13]. This fact, unfortunately, has led to an improper phraseology persisting in the literature indicating that spores have an “electron dense” outer layer (please see Appendix S1). Therefore, to facilitate interpretation of our results, we have included a line drawing of a conventional electron micrograph showing a thin section of a *Bacillus* spore in which the dark-staining outer coat is evident (Fig. 1*c*).

**Figure 2:**
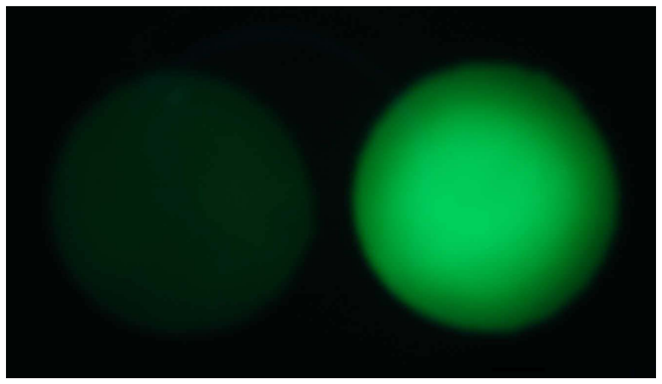
Germination-dependent fluorogenicity of F-spores. Image of two side-by-side glass fiber disks (6.35-mm diameter) each containing about 2.5×10^7^ F-spores of *B. megaterium* constructed with diacetylfluorescein. Left: Dormant F-spores. Right: F-spores exposed to germinant (4 mM D-glucose). Disks were prepared as indicated in Materials and Methods, and their fluorescence images were captured after incubation for 10 min at 37°C.

Using chemical analysis, we established that F-spores constructed with diacetyl-2,4,5,7-tetraiodo-fluorescein had an average of 1.8×10^6^ fluorogenic molecules per cell. Using this value and the mass of the electron-dense layer estimated from Fig. 2, we calculated the average decoration density to be one fluorogenic molecule per 6.2 kDa protein residue.

### Germination-Dependent Fluorogenicity of F-Spores

Initial experiments—using *B. megaterium* F-spores constructed with diacetylfluorescein—indicated that F-spores generate intense green fluorescence during germination (Fig. 2).

To ascertain that the fluorogenic activity of F-spores is under control of the germination mechanism, we used four different lines of experimentation: (i) germinant specificity; (ii) kinetics of germination; (iii) F-spores derived from germination-defective mutants; and (iv) effect of lethal conditions.

**Figure 3:**
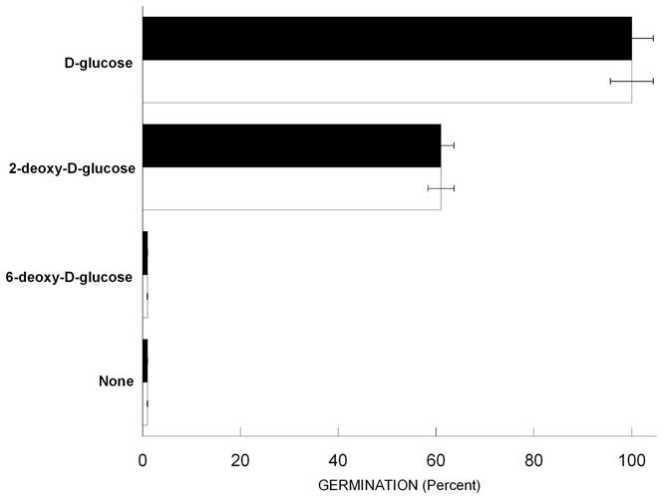
Germinant Specificity of F-spores. *B. megaterium* F-spores (constructed with diacetylfluorescein) were exposed to 4 mM of the indicated D-glucose derivatives at 37°C for 20 min. Fluorescence: Black bars. Optical density loss: White bars. Fluorescence and optical density loss were measured as indicated in Materials and Methods. Data were normalized using as 100% the values of F-spores induced to germinate by D-glucose. Error bars indicate SD of the mean.

To study germinant specificity, we constructed F-spores from *B. megaterium*, ATCC No. 14581, a strain producing spores that are specifically germinated by a family of monosacharides closely related to D-glucose. The response of F-spores to germinants was measured in terms of both fluorogenic ability and loss of optical density, a traditional parameter for quantitative measurements of germination rate based on rapid reduction of the spore's refractive index immediately after breaking dormancy [14], [15]. As shown in Fig. 3, fluorescence and loss of optical density were concomitantly induced by either D-glucose or 2-deoxy-D-glucose (both germinants of normal spores), whereas 6-deoxy-D-glucose (which is not an inducer) did not elicit either fluorescence or optical density loss. In addition, we established that other carbohydrates that are not germinants of normal spores invariably failed to induce germination of F-spores. These included D-fructose, D-maltose, methyl-ß-D-glucopyranoside, and methyl-α-D-glucopyranoside (data not shown).

Germinant specificity was also tested using F-spores constructed from different bacterial strains representing five species, *B. anthracis*, *B. atrophaeus*, *B. cereus*, *B. megaterium*, and *B. subtilis*. F-spores from each species representative were exposed to germinants known to be specific for the particular species, and their fluorogenic response was measured by the fluorescence produced after incubating the F-spores at 37°C for 20 min. The results showed that F-spores from each species were consistently induced to produce fluorescence by their corresponding specific germinants (Table 1). Quantitatively, the ratio between induced and baseline fluorescence (signal-to-noise ratio) was found to be consistently high (>400).

**Table 1:** Fluorogenic ability of F-spores constructed from different *Bacillus* spores.

F-spores^a^	Fluorescence (pixels/disk)^b^		S/N^c^
	Germinant^d^	None	
*B. anthracis*	91,917±14,171	213±138	432
*B. atrophaeus*	164,099±12,615	0	∞
*B. cereus*	41,925±2,246	19±13	2,207
*B. megaterium*	74,051±12,215	24±0	3,085
*B. subtilis*, PS533 (parental)	15,536±2,261	5±9	3,107
*B. subtilis*, FB72 (*ger-3*)	3±5	0	
*B. subtilis*, FB113 (*cwlJ sleB*).	3±5	0	
*B. megaterium* (killed)	0	0	

*^a^*F-spores were constructed using 3 mM diacetylfluorescein.

*^b^*Average fluorescence of triplicate measurements ± SD of the mean.

*^c^*S/N, signal-to-noise ratio.

*^d^*Germinants: For *B. anthracis* and *B. cereus*, 2.5 mM L-alanine together with 0.6 mM inosine—for *B. subtilis*, 1.1 mM L-alanine —for *B. megaterium*, 2.5 mM D-glucose—for *B. atrophaeus*, LB medium. Germination was induced at 37°C for 20 min.

**Figure 4:**
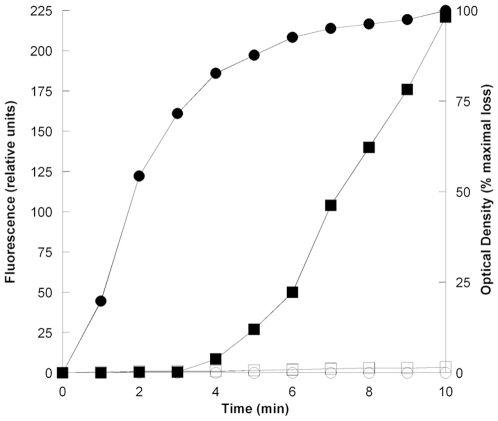
Germination kinetics of F-spores. *B. megaterium* F-spores (constructed with diacetylfluorescein) were induced to germinate by adding D-glucose (4 mM final concentration) at zero min, and fluorescence was measured at intervals as indicated in Materials and Methods. Black symbols: F-spores with germinant. White symbols: F-spores without germinant. Squares: Fluorescence. Circles: Optical density expressed as percentage of maximal loss (53%).

To determine germination kinetics of F-spores, we measured fluorescence and loss of optical density at short time intervals after inducing germination (Fig. 4). The results indicated that fluorescence has an initial lag period of approximately 3 min followed by a 10-min exponential increase. In these experiments, the loss of optical density of F-spores followed germination kinetics of normal spores, which typically show a brief lag, and a near maximal loss of approximately 50% occurring 10–15 min after inducing germination. The 3-min lag in fluorescence could be ascribed to activation of esterases during germination since it is comparable to the 4-min lag previously observed in germination kinetics of normal *B. anthracis* spores, which was measured by hydrolysis of extracellular diacetylfluorescein (compare Fig. 4 to Fig. 1 in reference [28]).

To analyze F-spores derived from germination-defective mutants, we constructed F-spores from two different mutants of *B. subtilis*, FB72 (*ger-A ger-B gerK*) and FB113 (*cwlJ sleB*). The former is impaired in three different germinant receptors [16], and the latter cannot degrade the spore cortex [17]. Our results showed that F-spores from these mutants—unlike F-spores from PS533, the parent strain—do not exhibit fluorogenicity (Table 1). This lack of fluorogenicity is probably associated with the germination defects because the mutant F-spores had an average of 1.3×10^6^ fluorogenic probes per F-spore as established by quantitative chemical analysis.

The effect of lethal conditions was tested by exposing *B. megaterium* F-spores to ethylene oxide for 16 h at a dose normally used for 100% killing of spores. We observed that ethylene oxide did not release fluorogenic probes from the F-spores (data not shown), but caused them to completely lose fluorogenic functionality (Table 1).

### Fluorogenicity of Individual F-Spores

**Figure 5:**
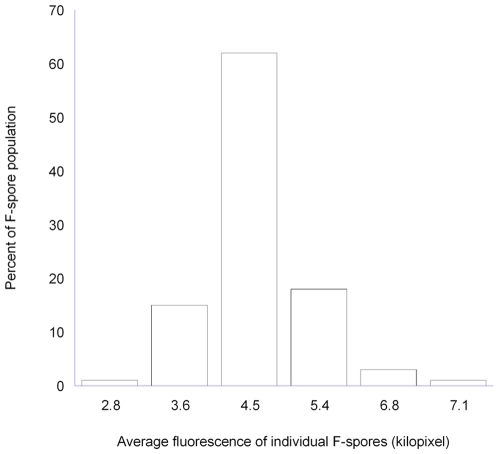
Distribution of fluorogenic functionality among F-spores. Histogram of fluorescence intensity among individual F-spores constructed with Syto 9. Fluorescent intensity was measured as indicated in Material and Methods.

To study distribution of fluorogenic functionality in individual F-spores, we used F-spores of *B. megaterium* (ATCC No. 14581) constructed with Syto 9. The reason for selecting Syto 9 is because its fluorescence increases significantly when bound to DNA or RNA [18], and therefore the fluorescent product should remain within the F-spores, whereas acyl esters of fluorescein generate water-soluble fluorescent products effluxing from germinated F-spores (data not shown). Specifically, germinated F-spores were placed on a microscope slide coated with agarose, and digital images were acquired using a fluorescence microscope. From the images, the fluorescence of single F-spores was quantitatively assessed using image analysis. The results (Fig. 5) indicate a normal distribution of fluorogenicity with average fluorescence intensity of 4,645 relative units per F-spore, and a median of 62% with average of 4,471 relative units per F-spore. A relatively small proportion (7%) of the population had no measurable fluorescence.

## Discussion

This work uncovers two unprecedented properties of coat proteins in *Bacillus* spores of various species. First, dormant spores spontaneously incorporate hydrophobic fluorogenic probes from the extracellular milieu, and use the probes to decorate a discrete layer of coat proteins. Second, the decorated layer causes spore fluorogenicity through a putative germination-dependent mechanism.

The adsorption of hydrophobic molecules by the coat can be rationalized since dormant spores have hydrophobic characteristics [19], [20]—probably due to the presence of hydrophobic coat proteins [21]—and have recently been shown to bind lypophilic dyes [22]. What is unexpected, however, is the topological specificity of the decorated layer indicating an unprecedented protein organization among coat proteins.

The layer is located near the surface of the outer coat (Fig. 1*a*), and it does not appear to include exosporium proteins (which may or may not be present in *B. megaterium* spores) because a similarly looking layer is observed in F-spores of *B. subtilis* (a genus without exosporium) constructed with fluorescein mercuric acetate, an electron-dense molecule (data not shown).

Before hypothesizing about the putative germination-dependent mechanism, we must take into account that Syto probes and fluorescein esters derive their fluorogenicity from entirely different biochemical pathways: Syto probes are weakly fluorescent molecules that significantly increase (e.g., 10–20 times) their fluorescence quantum yield by intercalating with DNA or RNA [18]; whereas fluorescein esters are non-fluorescent molecules producing intracellular fluorescent products when hydrolyzed by esterases [23].

**Figure 6:**
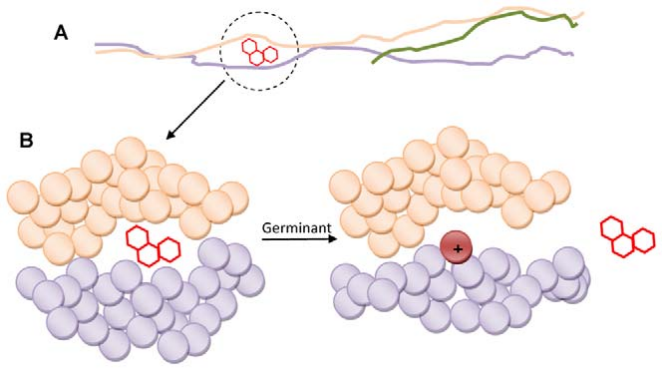
Schematic model of hydrophobic-hydrophilic transitions causing release of non-polar decorating fluorogenic molecules. The model shows how decorated coat proteins release a hydrophobic molecule by undergoing a germination-triggered hydrophobic-hydrophilic conformational change. Amino acid residues are depicted as balls and molecular sizes are not to scale. Disulfide bonds are not shown for simplicity. A. Cross-linked coat proteins become decorated by non-covalent binding of a non-polar fluorogenic molecule within a hydrophobic domain. B. During germination, the domain undergoes conformational changes causing a hydrophobic-hydrophilic transition (e.g., appearance of positive charges) and concomitant release of the fluorogenic molecule.

To account for the fluorogenicity of F-spores constructed with Syto 9, we propose a minimal mechanistic model schematically shown in Fig. 6. In the dormant F-spore, Syto 9 is constrained within hydrophobic domains of the decorated proteins (Fig. 6*a*). During early germination events, the decorated proteins undergo conformational changes leading to hydrophobic-hydrophilic transitions that promote the release of Syto 9. In Fig. 6*b*, the transition is shown as appearance of a positive charge. After release, Syto 9 is free to diffuse into the core where its fluorescence increases upon binding DNA and RNA. Precedents for postulating hydrophobic-hydrophilic transitions in proteins derive from studies of molecular chaperones [24], [25] and bacteriophage protein rearrangements [26], [27] in which an event—such as ligand binding or proteolysis—triggers hydrophobic-hydrophilic changes via conformational changes [28].

The model also explains the fluorogenicity of F-spores constructed with fluorescein esters since the germination-dependent hydrophobic-hydrophilic transitions in the decorated proteins would cause release of the bound ester molecules, which in turn diffuse in the cell and contact esterases (which are activated during germination) that hydrolyze them producing fluorescence [29]. It should be noted that fluorescein esters of short chain fatty acids, such as acetic and propionic acids, are hydrolyzed rather non-specifically by a broad family of intracellular enzymes including esterases, lipases, and proteases [30].

We should emphasize that implicit in the model is the assumption that the different hydrophobic fluorogenic probes used in this study behave much like diacetyl-2,4,5,7-tetraiodofluorescein. Indirect evidence supporting this assumption derives from competition experiments showing that fluorogenic probes displace each other from F-spores (data not shown). For example, diacetyl-2,4,5,7-tetraiodofluorescein was displaced 92% by an equal concentration of dibutyrylfluorescein.

Based on these results, we anticipate that F-spores constructed from coat protein-defective mutants will provide the means to identify particular proteins present within the decorated layer. In addition, we expect that F-spores constructed with functionally different probes will serve to monitor dynamic changes occurring among coat proteins soon after triggering germination, a research area remaining as an elusive target [1], [2]. For instance, F-spores constructed with fluorescein mercuric acetate—an electron dense fluorogenic substrate used as a probe that reacts specifically with sulfhydryl groups of proteins [31]—would serve a dual-purpose: to visualize molecular rearrangements of decorated proteins using TEM, and to quantitatively measure formation and breakage of disulfide bridges in space and time using fluorimetry.

Finally, the spore's ability to adsorb and specifically release hydrophobic molecules may find practical uses in emerging applications of spores as biosensors [32].

## Materials and Methods

### Bacterial Strains and Spore Preparation

The following bacteria were obtained from the American Type Culture Collection (ATCC, Manassas, VA): *B. atrophaeus* (ATCC 9372), *B. cereus* (ATCC 10876), and *B. megaterium* (ATCC 14581). *B. anthracis*, Sterne strain, was obtained from the National Veterinary Services Laboratory (Ames, IA). *B. subtilis*, strains PS533 (parent strain), FB72 (*ger-A gerB gerK*), and FB113 (*cwlJ sleB*) were generous gifts of P. Setlow (University of Connecticut Health Center, Farmington, CT). Bacteria were grown in Luria-Bertani medium at 30°C or 37°C, and spores were prepared and heat-activated as previously described [29].

### F-Spore Preparation

Two different methods were used for constructing F-spores. Most often, they were prepared in aqueous medium by mixing 200 µl of a spore suspension (10^9^ spores per ml) in 100 mM TRIS—20 mM NaCl, pH 7.4 (TRIS-NaCl) buffer with 5 µl of dimethyl sulfoxide containing a fluorogenic molecular probe at concentrations varying from 1.0 to 12 mM. The mixture was incubated at room temperature with occasional shaking for 15 min, and then the spores were separated by centrifugation at 16,200×*g* for 5 min at 4°C. After washing the spores twice with 200 µl cold TRIS-NaCl, they were resuspended in the same buffer and stored at 0°C for periods up to 48 h. Alternatively, for prolonged storage of F-spores in dry form, we used the following procedure. A 25-µl spore suspension (in sterile deionized water) was centrifuged in an acetone-resistant conical tube (Beckman polyallomer tube #357448) at 16,200×*g* for 5 min at 4°C, and the resulting pellet was dried under vacuum for 90 min. The dried spores were resuspended in 35 µl acetone containing a fluorogenic molecular probe at concentrations ranging from 1.0 to 12 mM. The spore suspension was dried under vacuum for 60 min and then stored over silica gel desiccant at room temperature. Under these conditions, F-spores were stable for periods up to 80 weeks. Prior to their use, F-spores were resuspended and washed twice in either TRIS-NaCl or 50 mM sodium phosphate, pH 7.25. It is notable that F-spores are indistinguishable from normal spores in cellular viability, heat resistance at 100°C, and refractivity under phase contrast microscopy.

The molecular probes used for constructing F-spores included: diacetyl-2,4,5,7-tetraiodofluorescein, diacetylfluorescein, dibutyrylfluorescein, dipropionylfluorescein, and 3-O-methyl-fluorescein acetate—synthesized as previously described [33]—fluorescein mercuric acetate (Fluka, Milwaukee, WI), Syto 9, Syto 16, Syto 21, Syto 23, and Syto BC (Invitrogen, Carlsbad, CA). Typically, F-spores had approximately 3×10^6^ fluorogenic molecules per cell, as determined by chemical analysis.

### Quantitative Assessment of Germination

Fluorescence of F-spores during germination was measured on glass fiber disks (6.35 mm diameter, GF/A Whatman, Piscataway, NJ) each receiving a 12-µl sample from a 40-µl reaction mixture containing TRIS-NaCl, 3×10^7^ spores, and germinant (e.g., L-alanine, D-glucose, and inosine) at concentrations varying from 0.2 to 2 mM. The disks were incubated in a moist chamber at 37°C for 10–20 min, and their fluorescence was quantitatively measured *in situ* using an image analyzer [34]. Triplicate measurements were made for each determination, and the average green fluorescence was expressed as number of fluorescent pixels within a circular region of 3,600 pixels in the center of each disk. Pixel data were not corrected for either threshold or baseline fluorescence, which was obtained from control disks without germinant. Generally, baseline fluorescence ranged from 0 to 350 pixels per disk. Kinetic experiments were performed by placing glass fiber disks containing reaction mixtures with F-spores on a thermostated heating block positioned under the image analyzer [29]. Loss of optical density during spore germination was measured as previously described [15].

### Cryosectioning and Electron Microscopy

Cryosectioning was selected because—in contrast to conventional sectioning using embedding resins—it does not require organic solvents that could remove fluorogenic probes from F-spores. Heat-activated spores of *B. megaterium* (ATCC No. 14581) were converted to F-spores as indicated above by exposure to TRIS-NaCl containing 13.2 µM diacetyl-2,4,5,7-tetraiodofluorescein, an electron dense fluorogenic substrate. F-spores suspended in phosphate-buffered saline were fixed in 2.5% glutaraldehyde (EM grade, Polysciences, Warrington, PA) at room temperature for 30 min, and then were separated by centrifugation at 16,200×*g* for 5 min at 4°C. After washing the spores twice with 200 mM glycine to eliminate excess glutaraldehyde, the spore pellet was covered with 2.3 M sucrose (without disturbing the pellet) and stored at 0°C overnight. A portion of the pellet was mounted on an aluminum pin, excess sucrose was removed with a filter paper, and the pin was plunged into liquid nitrogen. Ultrathin sections (60 nm) were cut at −120°C with a cryo-diamond knife. Sections were picked up from the knife with a loop dipped in 2.3 M sucrose and transferred to a formvar/carbon coated copper grid. Grids were floated on a drop of 2% methyl cellulose (in water) for 5 min, picked up with a loop and excess methyl cellulose was removed with a Whatman #1 filter paper to form an even layer of methyl cellulose over the grid (to avoid drying artifacts). Cryosections from normal spores were used as controls. Grids were examined by TEM using a Tecnai G° Spirit BioTWIN. We should note that cryosections were not stained.

### Distribution of Fluorogenic Ability among Individual F-Spores

F-spores of *B. megaterium* (ATCC No. 14581) constructed with Syto 9 were germinated by exposure to 1.6 mM D-glucose in TRIS-NaCl at 37°C for 20 min. The germinated F-spores were placed on a slide coated with agarose, and digital images were immediately captured using a fluorescence microscope (Zeiss, Thornwood, NY) equipped with a Kodak MDS 290 camera. From images showing about 200 F-spores, fluorescence of individual F-spores was quantitatively measured using image analysis software (Scanalytics, Rockville, MD). Briefly, two images of the same microscope field were collected using phase contrast and fluorescence microscopy. The phase contrast image served for locating individual spores, and the fluorescence image was used for quantitative measurements of fluorescence by determining the number of green fluorescent pixels in circular areas containing individual spores (areas without spores provided baseline fluorescence). Pixel data were not corrected for threshold, and the baseline fluorescence was zero.

## Supporting Information

Appendix S1 (0.04 MB RTF)

## References

[1] Moir A (2006). How do spores germinate?. J Appl Microbiol.

[2] Setlow P (2003). Spore Germination.. Curr Opinion Microbiol.

[3] Driks A (1999). *Bacillus subtilis* spore coat.. Microbiol Mol Biol Rev.

[4] Jagtap P, Michailidis G, Zielke R, Walker AK, Patel N (2006). Early events of *Bacillus anthracis* germination identified by time-course quantitative proteomics.. Proteomics.

[5] Piggot PJ, Losick R, Sonenshein AL, Hoch JA, Losick R (2002). Sporulation genes and inter-compartment regulation.. *Bacillus subtilis* and its closest relatives American Society for Microbiology.

[6] Westphal AJ, Price PB, Leighton TJ, Wheeler KE (2003). Kinetics of size changes of individual *Bacillus thuringiensis* spores in response to changes in relative humidity.. Proc Natl Acad Sci U S A.

[7] Plomp M, Leighton TJ, Wheeler KE, Hill HD, Malkin AJ (2007). In vitro high-resolution structural dynamics of single germinating bacterial spores.. Proc Natl Acad Sci U S A.

[8] Wang R, Krishnamurthy SN, Jeong JS, Driks A, Mehta M (2007). Fingerprinting species and strains of Bacilli spores by distinctive coat surface morphology.. Langmuir.

[9] Driks A (2003). The dynamic spore.. Proc Natl Acad Sci U S A.

[10] Chada VG, Sanstad EA, Wang R, Driks A (2003). Morphogenesis of *Bacillus* spore surfaces.. J Bacteriol.

[11] Henriques AO, Moran CPJ (2007). Structure, assembly, and function of the spore surface layers.. Annu Rev Microbiol.

[12] Kim H, Hahn M, Grabowski P, McPherson DC, Otte MM (2006). The *Bacillus subtilis* spore coat protein interaction network.. Mol Microbiol.

[13] Robinow CF (1953). Spore structure as revealed by thin sections.. J Bacteriol.

[14] Hachisuka Y, Asano N, Kato N, Okajima M, Kitaori M (1955). Studies on spore germination. I. Effect of nitrogen sources on spore germination.. J Bacteriol.

[15] Nicholson WL, Setlow P, Hardwood CR, Cutting SM (1990). Sporulation, germination and outgrowth.. Molecular biological methods for *Bacillus*.

[16] Paidhungat M, Setlow P (2000). Role of Ger-proteins in nutrient and non-nutrient triggering of spore germination in *Bacillus subtilis*.. J Bacteriol.

[17] Paidhungat M, Ragkousi K, Setlow P (2001). Genetic requirements for induction of germination of spores of *Bacillus subtilis* by Ca2+-dipicolinate.. J Bacteriol.

[18] Haugland RP, Spence MTZ, Johnson ID, Basey A (2005). A Guide to Fluorescent Probes and Labeling Technologies, 10 ed.

[19] Doyle RJ, Nedjat-Haiem F, Singh JS (1984). Hydrophobic characteristics of *Bacillus* spores.. Curr Microbiol.

[20] Wiencek KM, Klapes NA, Foegeding PM (1990). Hydrophobicity of *Bacillus* and *Clostridium* spores.. Appl Environ Microbiol.

[21] Zhang J, Fitz-James PC, Aronson AI (1993). Cloning and characterization of a cluster of genes encoding polypeptides present in the insoluble fraction of the spore coat of *Bacillus subtilis*.. J Bacteriol.

[22] Magge A, Setlow B, Cowan AE, Setlow P (2009). Analysis of dye binding by and membrane potential in spores of *Bacillus* species.. J Appl Microbiol.

[23] Rotman B, Papermaster BW (1966). Membrane properties of living mammalian cells as studied by enzymatic hydrolysis of fluorogenic esters.. Proc Natl Acad Sci USA.

[24] Swain JF, Dinler G, Sivendran R, Montgomery DL, Stotz M (2007). Hsp70 chaperone ligands control domain association via an allosteric mechanism mediated by the interdomain linker.. Mol Cell.

[25] Xu Z, Sigler PB (1998). GroEL/GroES: structure and function of a two-stroke folding machine.. J Struct Biol.

[26] Conway JF, Wikoff W, Cheng N, Duda R, Hendrix R (2001). Virus maturation involving large subunit rotations and local refolding.. Science.

[27] Leiman P, Chipman P, Kostyuchenko V, Mesyanzhinov V, Rossmann M (2004). Three-dimensional rearrangement of proteins in the tail of bacteriophage T4 on infection of its host.. Cell.

[28] Goh C-S, Milburn D, Gerstein M (2004). Conformational changes associated with protein–protein interactions.. Curr Opinion Struct Biol.

[29] Ferencko L, Cote MA, Rotman B (2004). Esterase activity as a novel parameter of spore germination in *Bacillus anthracis*.. Biochem Biophys Res Commun.

[30] Lavis LD (2008). Ester bonds in prodrugs.. ACS Chem Biol.

[31] Setlow P, Kornberg A (1969). Biochemical Studies of Bacterial Sporulation and Germination. XVII. Sulfhydryl and Disulfide Levels in Dormancy and Germination.. J Bacteriol.

[32] Ricca E, Cutting SM (2003). Emerging Applications of Bacterial Spores in Nanobiotechnology.. J Nanobiotechnology.

[33] Rotman B, Zderic JA, Edelstein M (1963). Fluorogenic substrates for beta-D-galactosidases and phosphatases derived from fluorescein (3,6-dihydroxyfluoran) and its monomethyl ether.. Proc Natl Acad Sci USA.

[34] Rotman B, MacDougall DE (1995). Cost-effective true-color imaging system for low-power fluorescence microscopy.. CellVision.

