# Supplementary material for: Constructing Fluorogenic Bacillus Spores (F-Spores) via Hydrophobic Decoration of Coat Proteins
Source: PLoS One. 2010 Feb 19;5(2):e9283. doi: 10.1371/journal.pone.0009283 (PMC2824812; doi:10.1371/journal.pone.0009283)
Supplement: Appendix S1 — (0.04 MB RTF) [file pone.0009283.s001.rtf]

Appendix S1
The misleading notion that the coat itself is “electron dense” appears to originate from descriptions of electron micrographs in the 1950s.  For instance, Robinow referred to a “dense outer coat” in spore sections fixed with osmium tetroxide (a commonly used electron-dense reagent) although there was no such structure in unfixed sections shown in the same paper [1].  Consequently, a preferable and factual description of the outer coat layer should be (as an example): “a dark-staining outer spore coat”[2].  In our work, glutaraldehyde-fixed unstained spores exhibited a relatively wide layer (about 150 nm) of medium electron density (Fig. 2b).  
References
1. Robinow CF (1953) Spore structure as revealed by thin sections. J Bacteriol 66: 300-311.
2. Ho-San K, Sherman D, Johnson F, Aronson AI (2004 ) Characterization of a major Bacillus anthracis spore coat protein and its role in spore inactivation. J Bacteriol 186: 2413-2417.
